# Hexagonal Zr_3_X (X = Al, Ga, In) Metals: High Dynamic Stability, Nodal Loop, and Perfect Nodal Surface States

**DOI:** 10.3389/fchem.2020.608398

**Published:** 2020-11-19

**Authors:** Heju Xu, Hailong Xi, Yong-Chun Gao

**Affiliations:** College of Science, North China University of Science and Technology, Tangshan, China

**Keywords:** nodal loop states, nodal surface states, first-principles, electronic structures, spin-orbit-coupling

## Abstract

In recent years, topological semimetals/metals, including nodal point, nodal line, and nodal surface semimetals/metals, have been studied extensively because of their potential applications in spintronics and quantum computers. In this study, we predict a family of materials, Zr_3_X (X = Al, Ga, In), hosting the nodal loop and nodal surface states in the absence of spin–orbit coupling. Remarkably, the energy variation of the nodal loop and nodal surface states in Zr_3_X are very small, and these topological signatures lie very close to the Fermi level. When the effect of spin–orbit coupling is considered, the nodal loop and nodal surface states exhibit small energy gaps (<25 and 35 meV, respectively) that are suitable observables that reflect the spin-orbit coupling response of these topological signatures and can be detected in experiments. Moreover, these compounds are dynamically stable, and they consequently form potential material platforms to study nodal loop and nodal surface semimetals.

## Introduction

The exploration of non-trivial topologies in crystalline solids has attracted significant attention from chemists, physicists, and material scientists (Kong and Cui, 2011; Cava et al., 2013; Banik et al., 2018; Zhang et al., 2018a; Tang et al., 2019). The main features of these topological solids are enclosed in their electronic-band structures. Initially, research was conducted in the context of the insulating state (Zhang et al., 2011; Li et al., 2012; Peng et al., 2012; Rasche et al., 2013; Wang et al., 2013; Kambe et al., 2014; Chang et al., 2015; Walsh et al., 2017; Barton et al., 2019; Zeugner et al., 2019), and the concept of band topology has now been extended to the metallic and semi-metallic states (Bradlyn et al., 2017; Bernevig et al., 2018; Schoop et al., 2018; Zhou et al., 2018; Gao et al., 2019; Hu et al., 2019; Klemenz et al., 2020; Wang et al., 2020b,c; Zhao Z. et al., 2020) as well.

The dimensionality of band-crossings is a criterion used to classify topological semimetals/metals. The most famous topological semimetals/metals with zero-dimensional band-crossings, i.e., zero-dimensional nodal points, are Dirac semimetals/metals (Chen et al., 2015, 2020; Bradlyn et al., 2017; Zhong et al., 2017; Jing and Heine, 2018; Liu et al., 2018b; Zhang et al., 2018b; Khoury et al., 2019; Wang et al., 2020f; Xu et al., 2020) and Weyl semimetals/metals (Peng et al., 2016; Lin et al., 2017; Fu et al., 2018; Zhang et al., 2018c; Zhou et al., 2019; Gupta et al., 2020; Jia et al., 2020; Liu et al., 2020; Meng L. et al., 2020; Zhao B. et al., 2020). We selected Weyl semimetals/metals as examples here because there exists a band-crossing of the valance band and conduction band at an isolated nodal point in the momentum space of these solids. Particularly, around this isolated nodal point, the quasiparticle acts similarly to the behavior of Weyl fermions, which are particles of considerable interest in high-energy physics. We summarize some recent studies on Weyl materials as follows: (i) Zhao and Ma (2020) stated that hexagonal MnO ferromagnet is a magnetic Weyl semimetal with spin-gapless state; (ii) Meng W. et al. (2020b) predicted that HfCuP compound is a newly designed Weyl semimetal with different types of Weyl nodes; and (iii) Jia et al. (2020) reported that the VI_3_ monolayer hosts a Weyl fermion and 100% spin-polarization. Furthermore, under the protection from certain crystalline symmetries, two Weyl points of opposite chirality can be stable at the same point, forming a Dirac point.

In the case of three-dimensional materials, besides the zero-dimensional nodal point metals/semimetals, in principle, there should exist one-dimensional and two-dimensional band-crossing metals/semimetals as well. For three-dimensional materials with one-dimensional band-crossings, some members, named as nodal line/loop semimetal/metals, have garnered considerable attention owing to their rich properties. Based on the shape of the nodal lines, they may host various forms, such as nodal link (Yan et al., 2017), nodal chains (Bzdušek et al., 2016), nodal boxes (Sheng et al., 2017), nodal ring (Wang et al., 2020d), nodal knot (Bi et al., 2017), and nodal net (Feng et al., 2018; Wang et al., 2018). So far, numerous types of nodal-line semimetals/metals have been predicted (He et al., 2019, 2020; Jin et al., 2019a,b, 2020a,c; Zhang et al., 2019; Meng W. et al., 2020a; Wang et al., 2020a,e; Zhou et al., 2020), and it is assumed that the node-line states have interesting characteristics in terms of their electronic, transport, and magnetic properties.

Zhong et al. (2016) had first observed topological semimetals/metals with two-dimensional band-crossings, i.e., nodal surface states. However, investigations into nodal surface semimetals/metals are very rare (Wu et al., 2018), and the energy variation of the nodal surface state is great.

If one material hosts two or more types of band-crossings, it can be considered a good platform to investigate the relationship among different topological signatures. Very recently, tetragonal PtO was proposed by Li et al. (2020b) as an effective material to study the one-dimensional nodal line and zero-dimensional nodal point states. Furthermore, Li and Xia (2020) predicted that cubic HfN is a topological material that co-exhibits nodal line and nodal loop states.

Motivated by the above-mentioned information and based on the first principles, we report a new family of topological materials, Zr_3_X (X = Al, Ga, In) with one-dimensional nodal loop and two-dimensional nodal surface states. The progress in the field of nodal line/surface states, including the conceptual development, the character and classification of these nodal structures, and the material realization, can be found in Wang et al. (2019). Moreover, the dynamical stable as well as the effect of spin-orbit coupling on the electronic structures of these materials are discussed in detail.

## Materials

**Figure 1:**
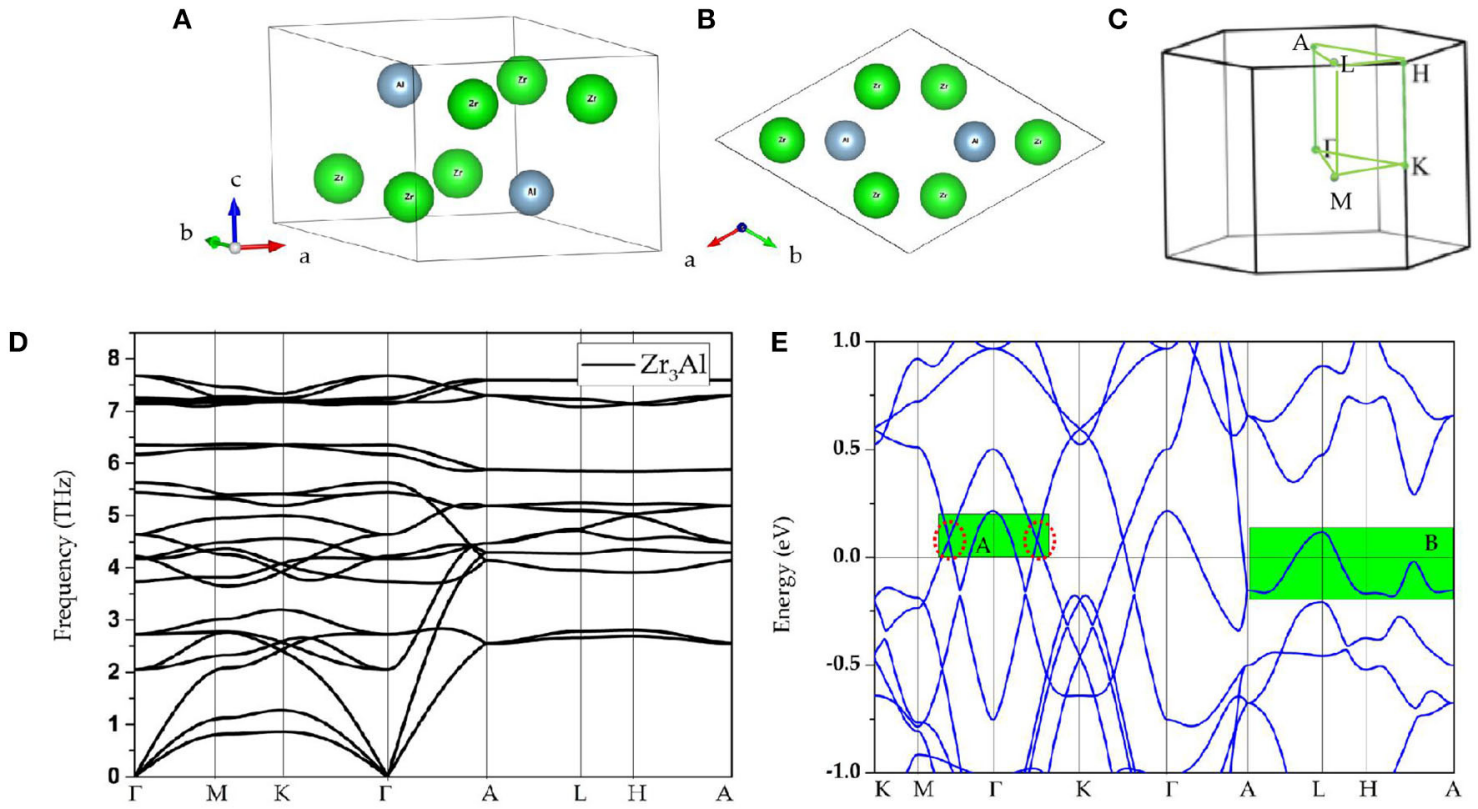
**(A,B)** Structures of Zr_3_Al as viewed from different sides; **(C)** Brillouin zone and the considered high-symmetry points; **(D)** calculated phonon dispersion of Zr_3_Al; **(E)** calculated band structure of Zr_3_Al; here, the obvious band-crossings can be found in region **A** and **B**.

In this study, we have focused on the hexagonal type Zr_3_X (X = Al, Ga, In). As an example, the primitive cell structure of hexagonal P6_3_/mmc type Zr_3_Al from different sides are shown in Figures 1A,B. From the figures, it is evident that Zr_3_X has eight atoms, namely, two X atoms and six Zr atoms. The structures of Zr_3_X have been totally relaxed with the help of first principles. The equilibrium lattice parameters of Zr_3_X (X = Al, Ga, In) have been computed via minimizing the crystal total energy calculated for different values of lattice constant by means of Murnaghan's equation of state (EOS) (Murnaghan, 1944). The achieved lattice constants for these compounds are shown in Table 1.

**Table 1:** Optimized lattice constants for Zr_3_X compounds.

**Compounds**	**a (Å)**	**b (Å)**	**c (Å)**
Zr_3_Al	6.202	6.202	5.371
Zr_3_Ga	6.166	6.166	5.052
Zr_3_In	6.325	6.325	5.221

**Figure 2:**
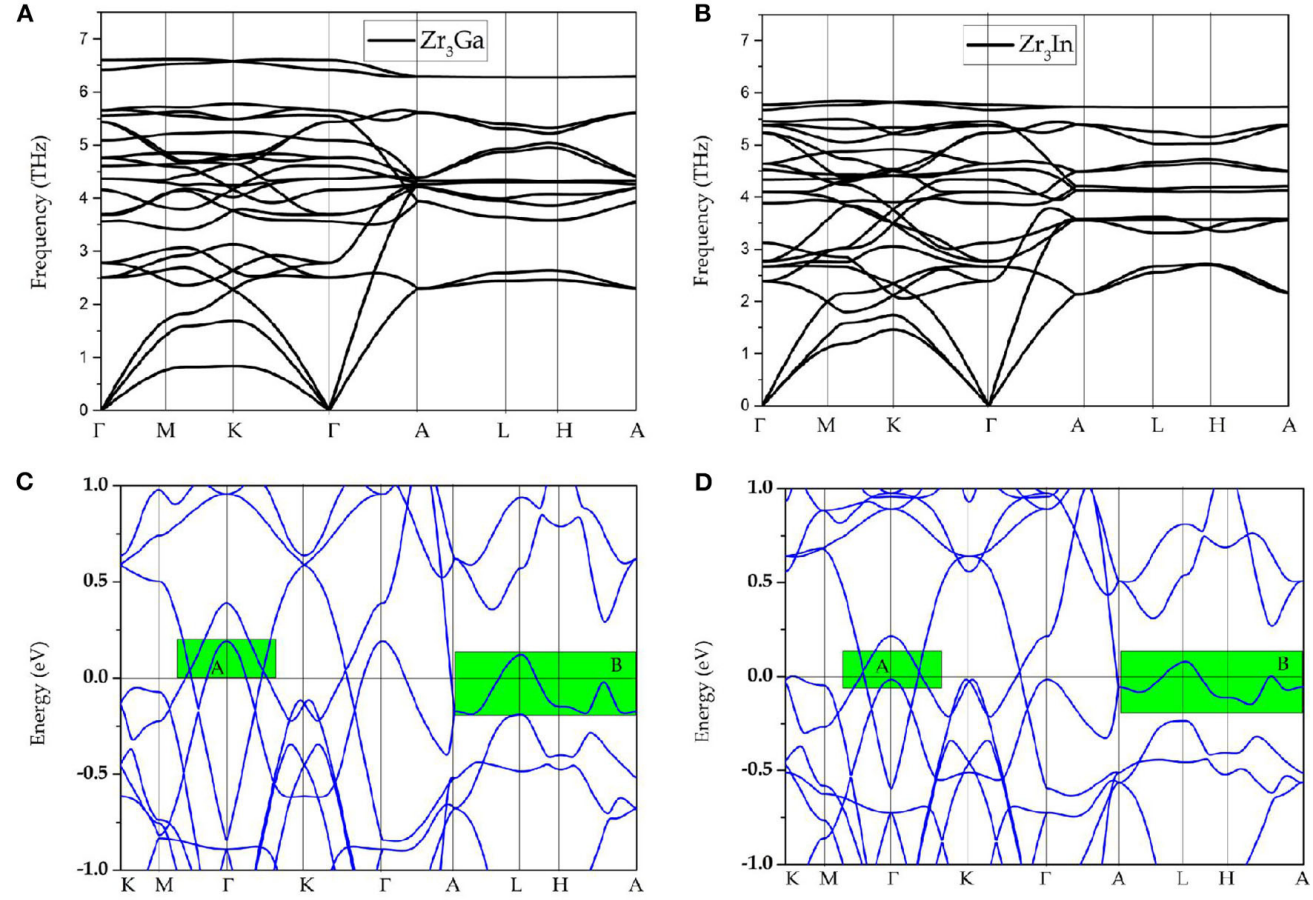
**(A,B)** Phonon dispersions of Zr_3_Ga and Zr_3_In, respectively; **(C,D)** calculated band structures of Zr_3_Ga and Zr_3_In, respectively; here, band-crossings around the Fermi level are highlighted by green rectangles.

Based on the Brillouin zone and considered high-symmetry points Γ-M-K-Γ-A-L-H-A (as shown in Figure 1C), dynamic stability was examined for these three compounds according to the calculated phonon dispersions, and the results are given in Figures 1D, 2A,B, respectively. These Zr_3_X compounds are obviously dynamically stable due to the absence of the imaginary frequency (Han et al., 2019; Wu et al., 2019; Li et al., 2020a). These materials are therefore proposed to be experimental platforms to study topological semimetals/metals.

## Computational Methods

In this study, calculations have been carried out using the Vienna ab initio simulation package (VASP) (Kresse and Furthmüller, 1996) based on the first-principles density functional theory (DFT), and the generalized gradient approximation (GGA) (Perdew et al., 1996) of Perdew–Burke–Ernzerhof (PBE) (Perdew et al., 1998) functional is adopted for the exchange-correlation potential. During the calculations, the cutoff energy is set as 600 eV, and the Brillouin zone is sampled by the Monkhorst–Pack *k*-mesh with a size of 6 × 6 × 6. Furthermore, we set the energy/force convergence criteria as 10^−6^ eV/10^−3^ eV.

## Results and Discussion

Observing the calculated band structure of Zr_3_Al in Figure 1E, we find that Zr_3_Al is a metal in which the bands and the Fermi level overlap. In addition to the metallic property, we find that there are several band-crossings near the Fermi level. These band-crossings are mainly located in two regions, named as region A and region B, which have been highlighted by green backgrounds. Similar properties are observed in Zr_3_Ga and Zr_3_In, as shown in Figures 2C,D, respectively.

In Figures 1E, 2C,D, one may notice that the band structures for these Zr_3_X compounds are approximately the same; hence, in the following discussion, Zr_3_Al was selected as an example with which to study the band topology of Zr_3_X compounds. As an example, the band structural of Zr_3_Al via GGA+U (U = 4 eV for Zr-d orbitals) is given in Supplementary Figure 1. One can find that the band topology of Zr_3_Al is still kept under GGA+U method.

In Figure 1E, we find that the band-crossings in region A and region B are quite close to the Fermi level. Specifically, the band-crossings in region A are along M-Γ-K paths, and the band-crossings in region B are along A-L-H-A paths. These band-crossings in both the regions may thus dominate the main features of Zr_3_Al.

**Figure 3:**
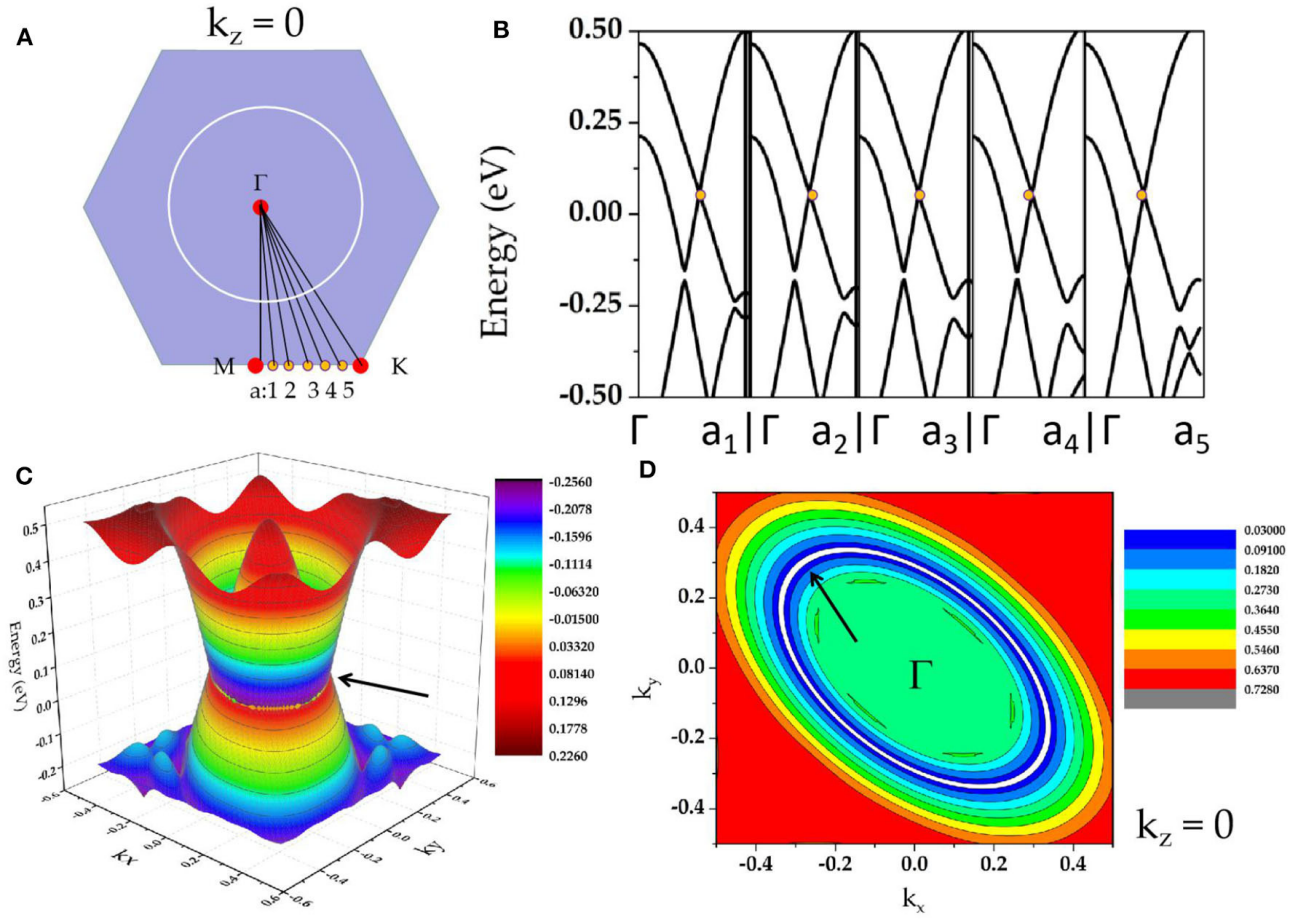
**(A)** Considered paths Γ-Q (Q = M, a_1_, a_2_, a_3_, a_4_, a_5_, K) in the k_z_ = 0 plane; **(B)** calculated band structures along Γ-a_1_, Γ-a_2_, Γ-a_3_, Γ-a_4_, Γ-a_5_ paths; **(C)** Γ-centered three-dimensional band dispersion in region A of the k_z_ = 0 plane; **(D)** shape of Γ-nodal line in region A of the k_z_ = 0 plane (the nodal line is shown as white lines and marked by arrows).

As shown in Supplementary Figure 1, in region A, we observe two obvious band-crossings; one is along M-Γ, and the other one is along Γ-K. Zr_3_Al is a system with inversion *P* and time-reversal *T* symmetries; thus, the two band-crossings along M-Γ-K paths cannot be isolated points (Xu et al., 2017; Fu et al., 2019) on the plane k_z_ = 0. To determine that the two band-crossings in region A belong to a nodal loop on the plane k_z_ = 0, we selected Γ-a_1_, Γ-a_2_, Γ-a_3_, Γ-a_4_, and Γ-a_5_ paths (see Figure 3A) to further calculate the band structures of Zr_3_Al (a_1_, a_2_, a_3_, a_4_, and a_5_ are equally spaced between M and K). The calculated band structures are shown in Figure 3B, and we find that band-crossings (marked as yellow circles) appear along Γ-a_1_, Γ-a_2_, Γ-a_3_, Γ-a_4_, and Γ-a_5_ paths, implying that a nodal loop should occur on the plane k_z_ = 0. The Γ-centered three-dimensional band dispersion in region A of the k_z_ = 0 plane and the shape of Γ-nodal line in region A are shown in Figures 3C,D, respectively. As shown in Figures 3B,C, these band-crossings in region A host very little energy variation. That is, Zr_3_X materials can be seen as exceedingly flat in energy, which may exhibit special properties that have exceptional applications. For example, very recently, Wang et al. (2020e) proposed that a nearly flat nodal line around the Fermi level will induce an exceptional thermoelectric power factor in the Nb_3_GeTe_6_ monolayer. Moreover, as shown in Figure 3B, we find that all the band crossing points on the plane k_z_ = 0 are type I (Liu et al., 2018a; see Supplementary Figure 2); this nodal line is thus type I.

**Figure 4:**
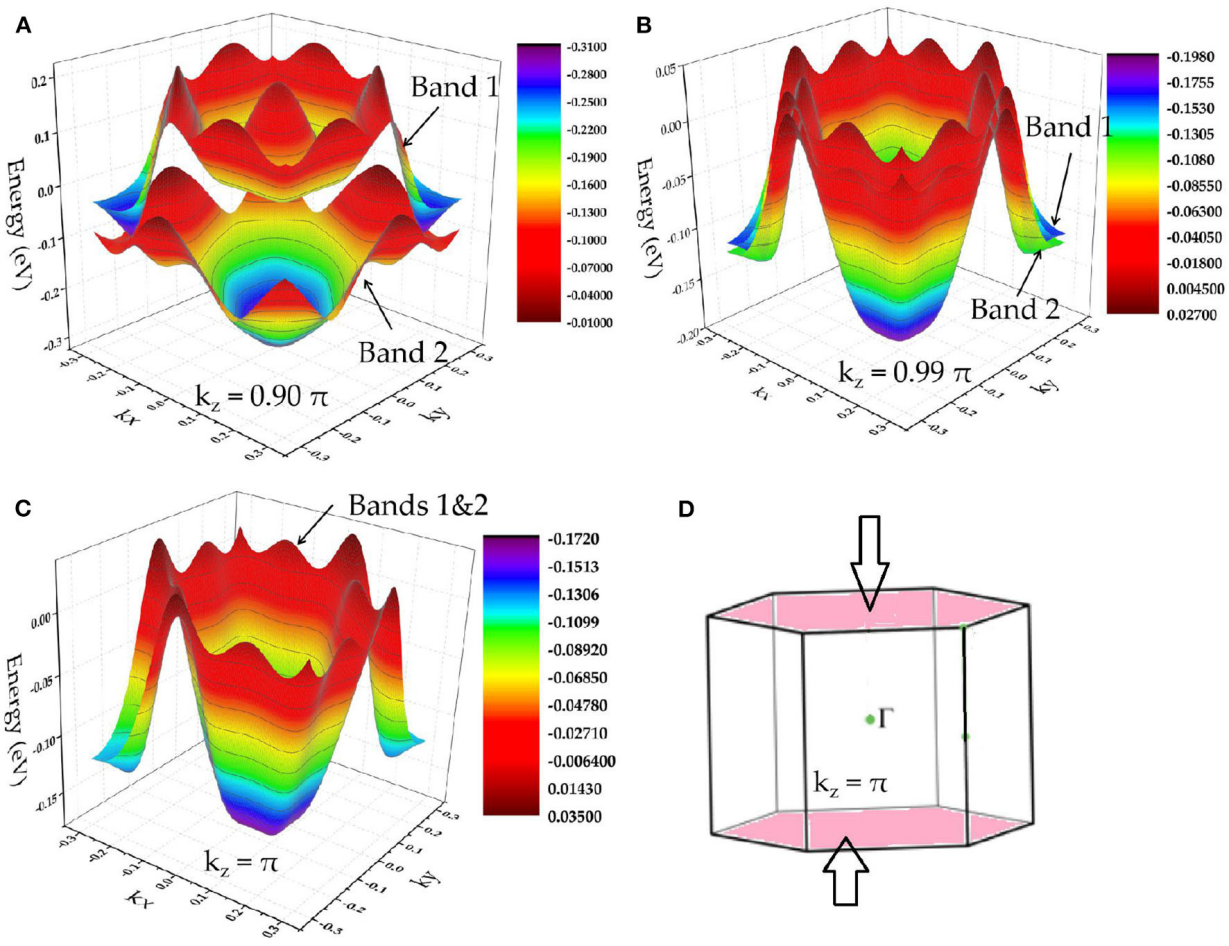
**(A–C)** A-centered three-dimensional band dispersion in region B of the planes k_z_ = 0.90 π, k_z_ = 0.99 π, and k_z_ = π, respectively; **(D)** schematic diagram indicating the nodal surface state in the k_z_ = π plane.

In region B, one can see that there are degenerate bands along the A-L-H-A direction. This indicates that the bands in the plane k_z_ = π are doubly degenerate, reflecting a nodal surface state that appeared in the plane k_z_ = π. To further confirm that the two bands are degenerated in k_z_ = π plane, we show the A-centered three-dimensional band dispersion in region B of the planes k_z_ = 0.90 π, k_z_ = 0.99 π, and k_z_ = π in Figures 4A–C, respectively. In Figure 4C, the two bands are obviously totally degenerate, leading to a new topological signature, i.e., nodal surface state, in the k_z_ = π plane (as shown in Figure 4D). Furthermore, as shown in Figure 4C, the energy variation of the nodal surface state is very small (range from −0.15 to 0.05 eV). Similar to the situation of the nearly flat nodal line state in the k_z_ = 0 plane, the small energy variation of the nodal surface state in the k_z_ = π plane may benefit the future experimental investigations.

**Figure 5:**
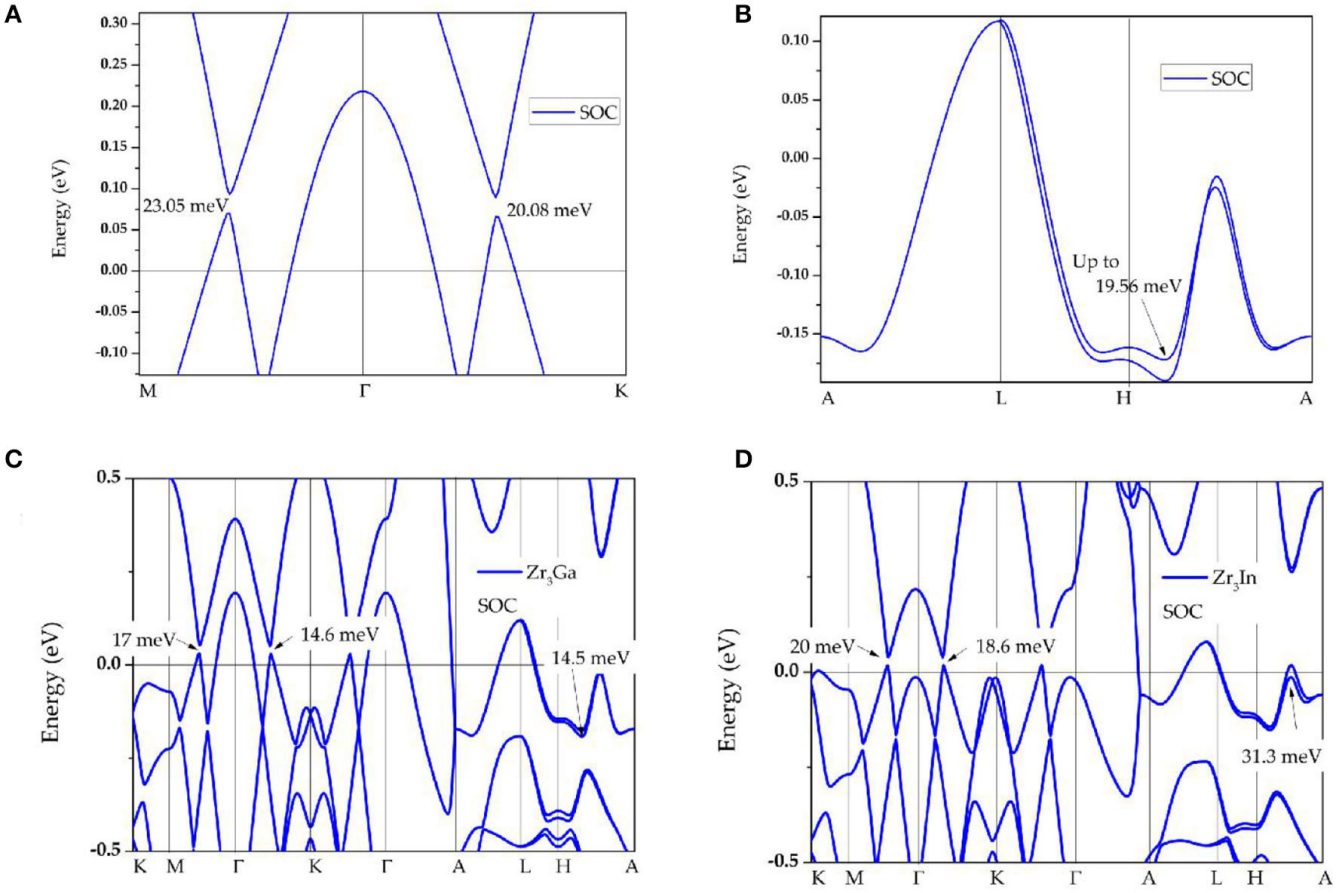
**(A,B)** Band structures of Zr_3_Al in the presence of spin–orbit coupling in region **A** and **B**, respectively; **(C,D)** band structures of Zr_3_Ga and Zr_3_In in the presence of spin–orbit coupling effect.

Finally, we discuss the electronic-band structure in the presence of spin–orbit coupling. The corresponding calculations results are shown in Figure 5. We find that Zr_3_Al is an excellent topological material whose band structure shows marked signatures (energy gaps) induced by the spin–orbit coupling effect (Fang et al., 2015). The spin–orbit coupling effect induces energy gaps of 23.05 and 20.08 meV (see Figure 5A) in region A. Furthermore, the band-crossings in region B have open energy gaps of 19.56 meV resulting from the spin–orbit coupling effect (see Figure 5B). The band structures of Zr_3_Ga and Zr_3_In with the effect of spin–orbit coupling are also exhibited in Figures 5C,D, respectively. The open energy gaps observed in these topological signatures, exhibited by Zr_3_X metal (X = Al, Ga, In), are very small compared to the other well-known topological semimetals/metals (Fang et al., 2016). A detailed collection of SOC gaps of typical nodal line materials can be found in the Supplementary Information of (Jin et al., 2020b).

## Summary

In summary, the topological band structures of Zr_3_X (X = Al, Ga, In) have been studied via DFT calculations in this study. Neglecting spin-orbit coupling, there is a nodal loop in the k_z_ = 0 plane and nodal surface state in the k_z_ = π plane. The rich topological signatures are quite near to the Fermi level, which can be detected experimentally. Remarkably, the loop is nearly flat and the nodal surface features small energy variation. These above-mentioned topological signatures are not sensitive to the effect of spin–orbit coupling. Further, these compounds are proved to be dynamically stable based on the calculated phonon dispersions and host simple and clear band structures. It is expected that these non-trivial band-crossings can be experimentally observed via angle-resolved photoemission spectroscopy (ARPES).

## Data Availability Statement

The original contributions generated for the study are included in the article/Supplementary Materials, further inquiries can be directed to the corresponding author/s.

## Author Contributions

HXu: investigation and writing-original draft. Y-CG: supervision. HXu and Y-CG: formal analysis. HXi: methodology. All authors contributed to the article and approved the submitted version.

## Conflict of Interest

The authors declare that the research was conducted in the absence of any commercial or financial relationships that could be construed as a potential conflict of interest.
