# Supplementary material for: Hexagonal Zr3X (X = Al, Ga, In) Metals: High Dynamic Stability, Nodal Loop, and Perfect Nodal Surface States
Source: Front Chem. 2020 Nov 19;8:608398. doi: 10.3389/fchem.2020.608398 (PMC7710705; doi:10.3389/fchem.2020.608398)
Supplement: Supplementary file 1 [file Data_Sheet_1.doc]

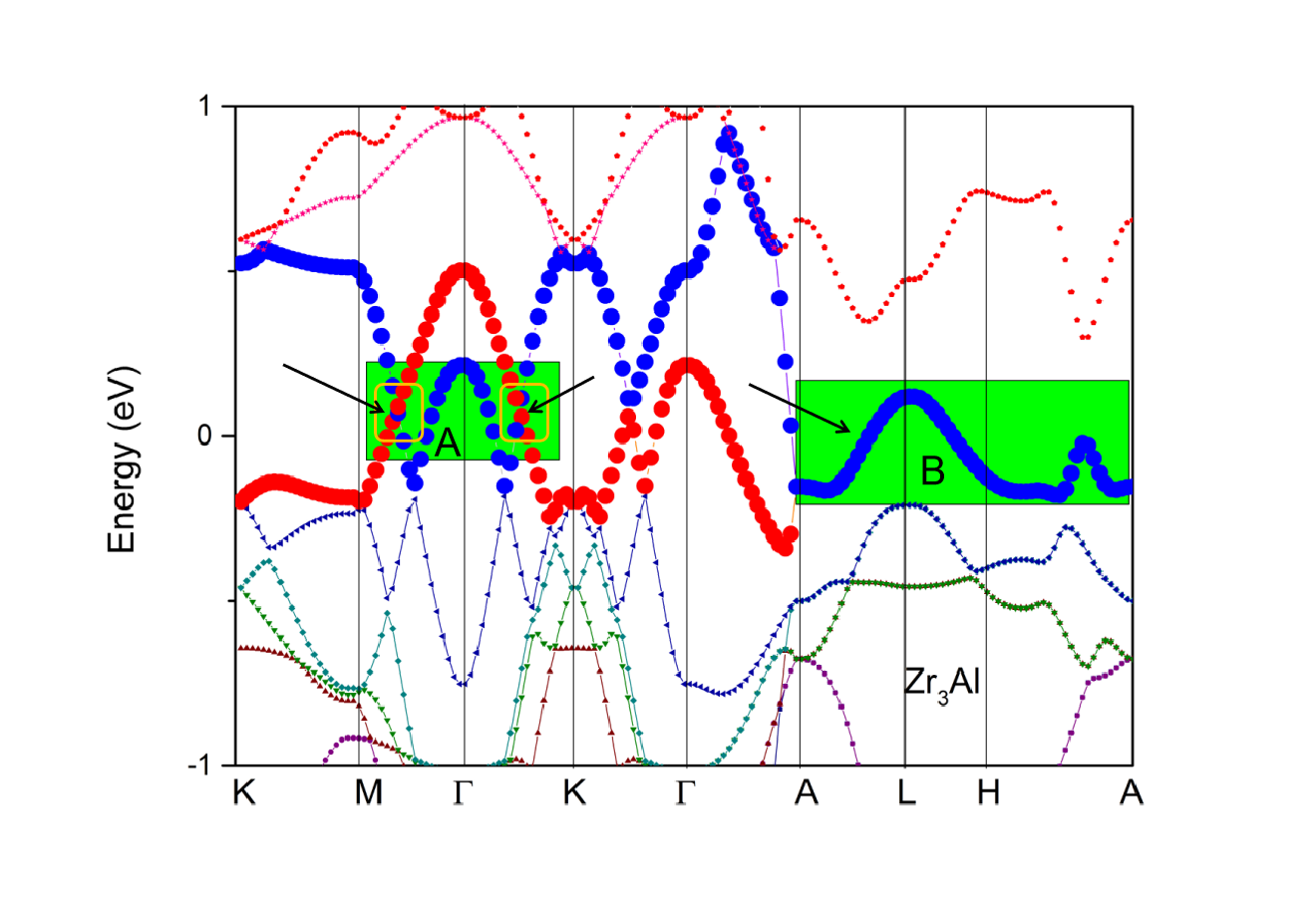


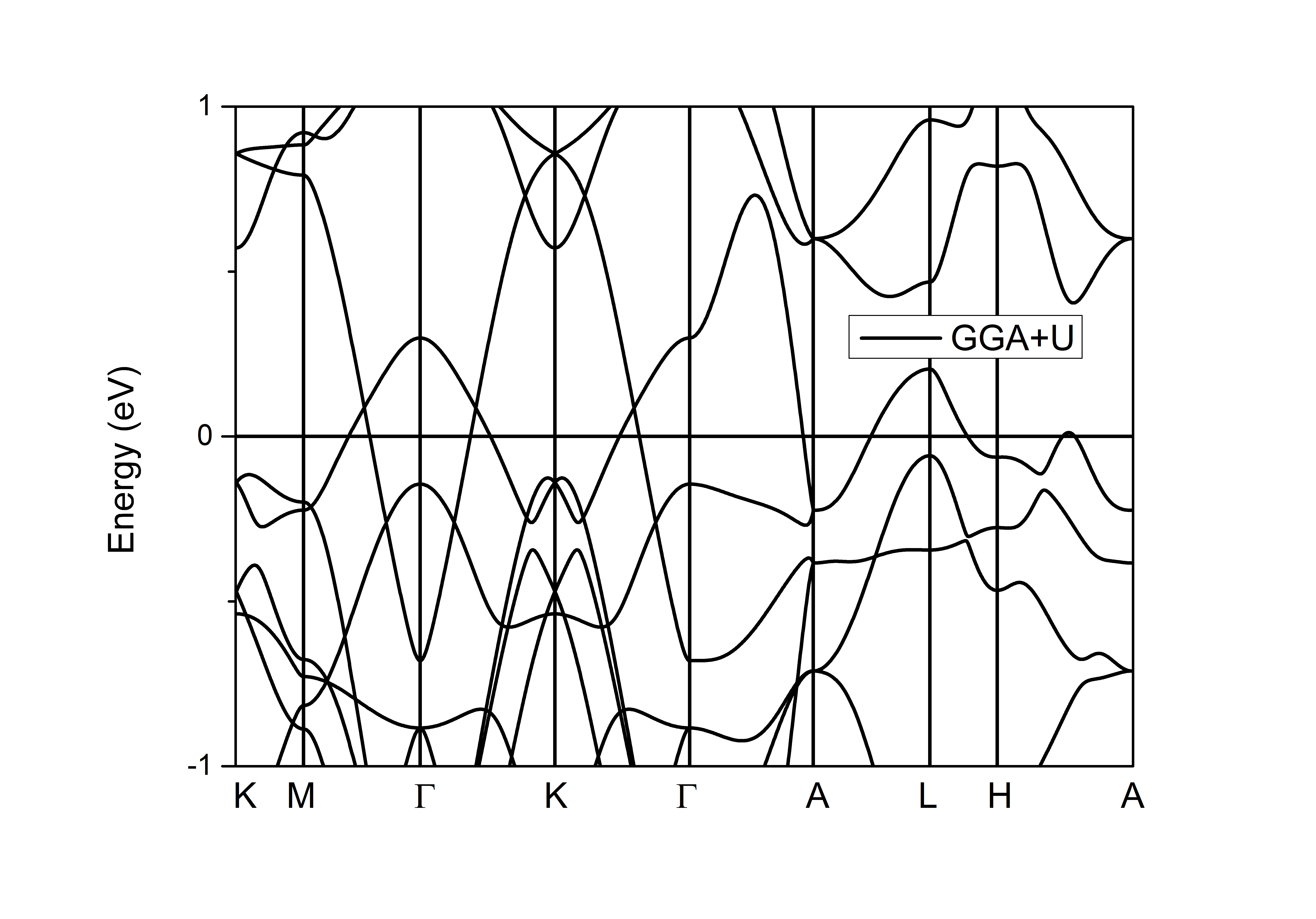


Figure S1: (upper) Band structure of Zr_3_Al with lines in colors; (bottom) Band structure of Zr_3_Al via GGA+U (U = 4 eV for Zr -d orbitals).


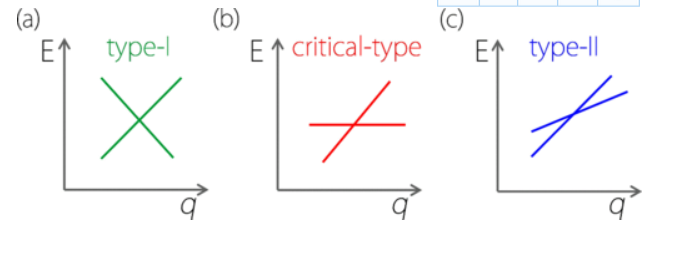


Figure S2: Schematic figure of type-I, critical-type, and type-II band dispersions in the momentum-energy space. For type-I band crossing points, the bands manifest conventional conical dispersions, with electron and hole regions well separated by the energy; For type II band crossing point, the spectrum is completely tipped over, which can give rise to the coexistence of electron and hole states at a given energy; For critical-type band crossing point, it is composed of a flat band and a dispersive band.
